# R2R3 MYB Transcription Factor GhMYB201 Promotes Cotton Fiber Elongation via Cell Wall Loosening and Very-Long-Chain Fatty Acid Synthesis

**DOI:** 10.3390/ijms25179559

**Published:** 2024-09-03

**Authors:** Qingwei Suo, Nianjuan Fang, Jianyan Zeng, Fulin Yan, Xi Zhu, Yi Wang, Wanting Yu, Junmin Chen, Aimin Liang, Yaohua Li, Jie Kong, Yuehua Xiao

**Affiliations:** 1 Chongqing Key Laboratory of Crop Molecular Improvement, College of Agronomy and Biotechnology, Southwest University, Chongqing 400715, China; suoqw0302@email.swu.edu.cn (Q.S.); fangnianjuan@163.com (N.F.); zengjianyan@swu.edu.cn (J.Z.); yflchina@hotmail.com (F.Y.); zhuxi0603@email.swu.edu.cn (X.Z.); norman@email.swu.edu.cn (Y.W.); yuwanting2024@163.com (W.Y.); chendajun@163.com (J.C.); liam2855@163.com (A.L.); 2 Institute of Economic Crops, Xinjiang Academy of Agricultural Sciences, Urumqi 830091, China; liyaohua61@163.com (Y.L.); kongjie.258@163.com (J.K.)

**Keywords:** *Gossypium hirsutum*, R2R3 MYB transcription factor, fiber elongation, cell wall loosening, KCS

## Abstract

Cotton fiber is the leading natural textile material, and fiber elongation plays an essential role in the formation of cotton yield and quality. Although a number of components in the molecular network controlling cotton fiber elongation have been reported, a lot of players still need to be functionally dissected to understand the regulatory mechanism of fiber elongation comprehensively. In the present study, an R2R3-MYB transcription factor gene, *GhMYB201*, was characterized and functionally verified via CRISPR/Cas9-mediated gene editing. GhMYB201 was homologous to *Arabidopsis* AtMYB60, and both coding genes (*GhMYB201At* and *GhMYB201Dt*) were preferentially expressed in elongating cotton fibers. Knocking-out of *GhMYB201* significantly reduced the rate and duration of fiber elongation, resulting in shorter and coarser mature fibers. It was found that GhMYB201 could bind and activate the transcription of cell wall loosening genes (*GhRDLs*) and also β-ketoacyl-CoA synthase genes (*GhKCSs*) to enhance very-long-chain fatty acid (VLCFA) levels in elongating fibers. Taken together, our data demonstrated that the transcription factor GhMYB201s plays an essential role in promoting fiber elongation via activating genes related to cell wall loosening and VLCFA biosynthesis.

## 1. Introduction

Cotton produces the majority of natural textile fibers in the world. Cotton fibers are extremely elongated unicellular structures developed from the outermost ovule epidermal cells through four distinct but overlapping stages: initiation, elongation (primary cell wall synthesis), secondary cell wall synthesis, and maturation [1,2,3]. The initiation of fibers usually undergoes from −3 to 3 days post-anthesis (DPA), with 20–30% of epidermal cells bulging out and finally differentiating into long fibers or lint [4]. Once initiated, fiber cells undergo rapid elongation from 0 to 20 DPA and reach the final length at 25–30 DPA. Cellulose begins to deposit at around 16 DPA and continues till 40–50 DPA. At the maturation and boll opening stage, fibers are dehydrated and collapsed, with a thick secondary cell wall composed of nearly pure cellulose [5]. The elongation stage determines the final length of cotton fibers, which is one of the most important indices of cotton fiber. Meanwhile, length is also a determinant of the weight of a single fiber, thus affecting yield. Notably, slowing down fiber elongation generally is coupled with the onset of the secondary cell wall synthesis stage and further influences fiber cell wall thickening and final quality [5,6].

Cotton fiber is one of the longest plant cells with a length-to-diameter ratio of up to 2000. Fast and polar linear elongation of cotton fiber is characteristic of vigorous expansion of the primary cell wall in the growing tip [3]. This complex dynamic process comprises the synthesis, transport, and deposition of cell wall components, remodeling of membrane and cell structures, and also redirection of cell metabolism and organization [7]. Numerous regulatory paths, including transcription factors, plant hormones, signaling small molecules, and structural proteins, have been reported to play roles in fiber elongation [7,8,9,10]. For example, two cell wall loosening proteins, GhRDL1 and GhEXP1, promote fiber elongation [11]. Saturated very-long-chain fatty acids (VLCFAs) enhance fiber elongation by activating 1-aminocyclopropane-1-carboxylic acid (ACC) oxidases (GhACOs) expression and ethylene biosynthesis [12], and its biosynthesis responses to gibberellin (GA), brassinosteroid (BR), and strigolactone (SL) signals via key β-ketoacyl-CoA synthase (KCS) genes [9,13,14].

Accumulating research reported the essential roles of transcription factors in regulating fiber elongation. Transcription factors generally function as key signaling components of phytohormones and/or direct activators/suppressors to modulate the transcription of structural genes. Transgenic cotton overexpressing BRI1-EMS-SUPPRESSOR1 (BES1), a positive BR-signaling transcription factor, produced significantly longer fibers, while its inhibition resulted in shorter fibers [14]. GhHOX3 and GhHOX4, two homeodomain-leucine zipper (HD-ZIP) transcription factors, both positively regulate fiber elongation [8,15]. Overexpression of PACLOBUTRAZOL RESISTANCE 1 (GhPRE1), a basic helix–loop–helix (bHLH) transcription factor, resulted in longer fibers [16]. Cotton DELLA protein GhSLR1, the major repressor in the GA signaling pathway, inhibited fiber elongation, while its interacted transcription factors GhHOX3, GhZFP8, and GhBLH1 promoted fiber elongation [9,15]. An appropriate ABA level may promote ethylene biosynthesis and fiber elongation by activating the expression of GhACO3 through the key ABA signaling transcription factor GhbZIP27a, which is preferentially expressed in the elongating fibers [17]. GhMYB25-silenced cotton altered the timing of fiber elongation, leading to short fibers [18]. GhMYB109 was important for fiber elongation, and silencing GhMYB109 resulted in shorter fibers [19]. GhMYB212 directly regulates the expression of sucrose transporter GhSWEET12, transporting sucrose into expanding fibers [20]. GhWRKY16 participates in fiber elongation by directly regulating the expression of *GhMYB25*, *GhHOX3*, *GhMYB109*, and cellulose synthase gene *GhCesA6D-D11* [4]. Recently, GhMYB86 was found to negatively affect fiber elongation by directly activating a tubulin gene *GhTUB7* [21]. Nevertheless, plenty of transcription factor genes significantly expressed in elongating fibers remained functionally characterized, and more work was necessary to comprehensively clarify the molecular network regulating cotton fiber elongation.

In this study, we identified an R2R3-MYB transcription factor GhMYB201 that is specifically expressed in the cotton fiber rapid elongation stage. Knocking out *GhMYB201* significantly decreased the fiber length. Further study revealed that GhMYB201 promoted fiber elongation by directly activating the expression of cell wall loosening genes (*GhRDLs*) and very-long-chain fatty acid synthase genes (*GhKCSs*). Our results provide a new insight into the molecular mechanism regulating cotton fiber elongation by revealing the function of GhMYB201, a positive regulator that plays a vital role in cotton fiber development.

## 2. Results

### 2.1. GhMYB201 Is a Transcriptional Activator Preferentially Expressed in Elongating Fibers

**Figure 1:**
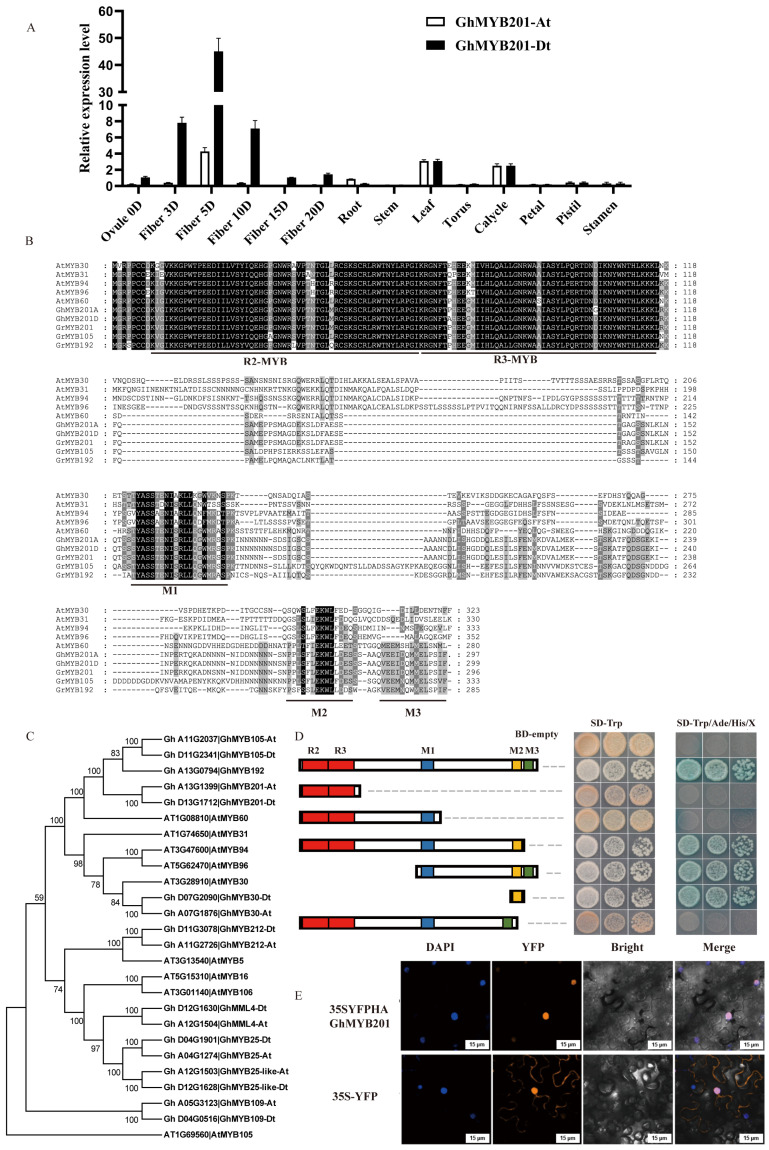
Characterization of *GhMYB201*. (**A**) The expression pattern of *GhMYB201At* and *GhMYB201Dt* in various tissues, organs, and fibers was detected by qRT-PCR. Ovule 0D: 0 DPA fibers with ovule; Fiber 3D: 3 PDA fibers without ovule; Fiber 5D: 5 PDA fibers without ovule; Fiber 10D: 10 PDA fibers without ovule; Fiber 15D: 15 PDA fibers without ovule; Fiber 20D: 20 PDA fibers without ovule. (**B**) The multi-sequence alignment of GhMYB201 and its homologs. Sequences were aligned by ClusterW. The conserved domain sites are highlighted, black part is strong and grey part is slightly weak. (**C**) Phylogenetic analysis of GhMYB201 and its homologs. The phylogenetic tree was constructed by the neighbor-joining method and tested using 1000 replicates of bootstrap. (**D**) Assay of GhMYB201 transcriptional activation activity in yeast. Full-length and truncated GhMYB201 were fused to the GAL4 DNA-binding domain and transformed into a Y2H strain. Strains harboring BD vectors could survive on the dropout medium (SD-Trp). The survival on the dropout medium (SD-Trp/His/Ade medium) supplied with X-α-gal indicated that the activation domain is located in the C terminal, probably the M2 domain. (**E**) Subcellular localization of GhMYB201 protein in leaf cells of *Nicotiana benthamiana*. 35S-YFP was used as a control. 4′,6-diamidino-2-phenylindole (DAPI) staining was used to stain the nuclei of tobacco. Bars = 15 μm.

Based on the previously published transcriptomic data [22], 24 transcription factor genes preferentially expressed in elongating fibers (enrichment fold > 3 and enrichment factor > 50) were identified (Table S1). Among them, two homologous GhMYB201 genes (*Gh_D13G1712* and *Gh_A13G1399*, named *GhMYB201Dt* and *GhMYB201At*, respectively) had the highest enrichment factors, and qRT-PCR analysis indicated that both *GhMYB201* genes were preferentially expressed in elongating fibers, with maximum expression levels in 5 DPA fibers (Figure 1A and Figure S1). GhMYB201s shared high similarity with *Arabidopsis* AtMYB60, conserved R2 and R3-MYB domains, and M1, M2, and M3 motifs (Figure 1B). Phylogenetic analysis indicated that GhMYB201s and closely related GhMYB105 and GhMYB192 [23] were homologous to AtMYB60 (Figure 1C), different from previously reported R2R3-MYB proteins involved in the regulation of the fiber initiation and growth, such as GhMYB25 [18], GhMYB25-like [24], GhMYB109 [19], GhMYB212 [20], and GhMYB30 [25].

When transformed into yeast, GhMYB201 fused with the GAL4 DNA-binding domain, which exhibited strong transcriptional activation activity on the downstream marker genes (Figure 1D). The following domain truncation analysis indicated that the transcriptional activation activity was due to the M2 motif conserved in the AtMYB60 group and the related AtMYB30 group (Figure 1C,D). Furthermore, GhMYB201 was fused to yellow fluorescent protein (YFP) and transiently expressed in tobacco leaves. Based on the overlapping signal of the YFP signal and 4′,6-diamidino-2-phenylindole (DAPI) staining, the YFP-HA-GhMYB201 protein was exclusively localized in the nucleus (Figure 1E). Consistent with sequence and expression analysis, these data suggested that GhMYB201s functioned as transcriptional activators in elongating cotton fibers.

AtMYB60 is involved in the transcriptional regulation of stomatal movements in *Arabidopsis*, and its null mutant (*atmyb60-1*) led to a constitutive reduction of stomatal opening [26,27]. To explore the biological function of GhMYB201, *GhMYB201* was overexpressed in *Arabidopsis* (Figure S2B). Under the same growth condition, the *GhMYB201* overexpressing leaves showed a significant increase in stomatal diameter compared to wild type (WT), in contrast to the *atmyb60-1* mutant (Figure S2A,C). This observation suggested that *GhMYB201* was a functional homolog of *Arabidopsis AtMYB60*.

### 2.2. GhMYB201 Knockout Negatively Affected Fiber Elongation

**Figure 2:**
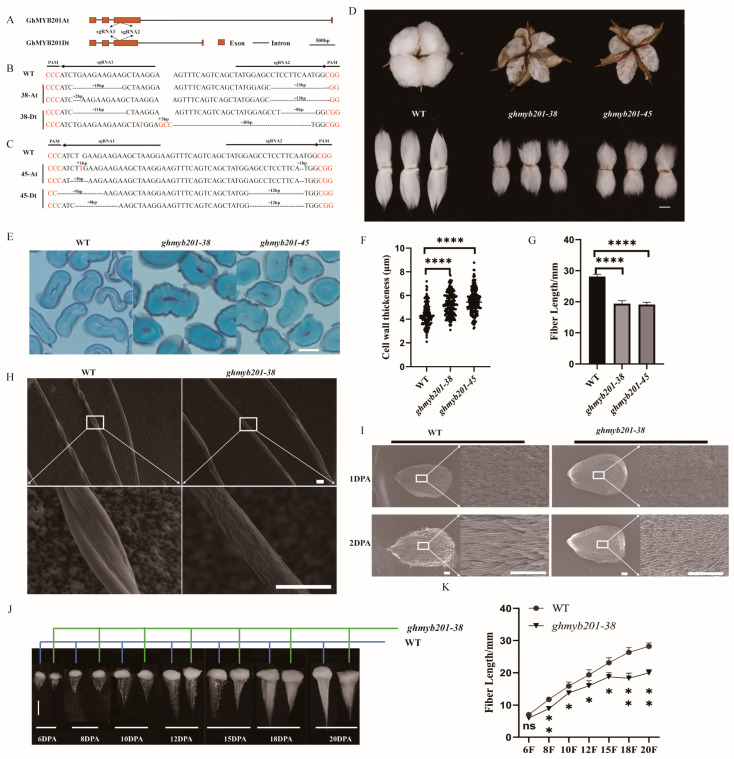
Phenotypic assay of fibers of GhMYB201 transgenic lines. (**A**) Diagram of CRISPR/Cas9 targeted sites on chromosomes A13 and D13. The *GhMYB201At* and *GhMYB201Dt* are illustrated with exon regions in brown boxes and intron regions with horizontal lines. Two guide RNA sequences were designed on the third exon. (**B**,**C**) Characterization of *GhMYB201* knockout lines #38 and #45 by Sanger sequencing in the T1 generation. WT indicates the unedited sequence. Horizontal lines denote the guide sequence region specific to GhMYB201, and the red bases denote the PAM recognition sites. Lines #38 and #45 both edited all the four chromosomes. (**D**) Image of mature cotton bolls and fibers of the wild type and lines #38 and #45. Bars = 10 mm (**E**) Photomicroscopics of the cross-sections of mature fibers from the wild type and lines #38 and #45. Bar = 10 μm. (**F**) Fiber cell wall thickness of the *GhMYB201* knockout lines and the wild type (*n* ≥ 100). Data are presented as the means ± SD. **** *p* < 0.0001 (Student’s *t*-test). (**G**) The mature fiber length of *GhMYB201* knockout lines and the wild type (*n* ≥ 30). Data are presented as the means ± SD. **** *p* < 0.0001 (Student’s *t*-test). (**H**) Scanning electron microscopy images of mature fibers from the wild type and line #38 with different magnification. Bars = 30 μm (**I**) Scanning electron microscopy images of ovule from the wild type and line #38. Bars = 200 μm (**J**) The Fiber length of the wild type and line #38 at fiber development stages of 6, 8, 10, 12, 15, 18, and 20 DPA. Error bars showed the SD of 3 biological replicates. Bar = 10 mm. (**K**) Comparison of fiber length at 6, 8, 10, 12, 15, 18, and 20 DPA from the wild type and line #38. Data are presented as the means ± SD. * *p* < 0.05, ** *p* < 0.01, ns means no statistically significant difference (Student’s *t*-test).

To explore the biological functions of GhMYB201s in fiber development, we generated stable *GhMYB201* knockout mutants using CRISPR/Cas9-mediated genome editing. Several independent knockout lines showing similar phenotypic variations were obtained. Two of them (*ghmyb201-38* and *-45*) were chosen for full characterization. At the guide RNA-targeted sites located in the third exon of *GhMYB201s*, lines #38 and #45 carried mutants in all four chromosomes, causing shifting or Indel (Figure 2A–C and Figure S4) and probably disrupting all the functional GhMYB201 proteins.

Instead of fluffy fibers observed in wild type opening bolls, mature fibers of the *GhMYB201* knockout mutants were tightly attached around seeds, somewhat like dead locules (Figure 2D). Unlike the traditional dead locule, e.g., immature fiber mutant [28], which resulted from incomplete development of secondary cell wall, *GhMYB201* knockout fibers had thicker secondary cell wall (Figure 2E,F) and significantly increased micronaire value (Table 1) compared to wild type. In addition, scanning electron microscopy (SEM) observation indicated that *GhMYB201* knockout fibers had more round and rough appearance and less conversion (Figure 2H). Finally, mature fiber length was significantly decreased in *GhMYB201* knockout lines compared with the wild type (Figure 2D,G, Table 1). We further compared the development dynamics between the *GhMYB201* knockout line and wild type cotton. It was found that the fiber length of the *GhMYB201* knockout line was significantly shorter than that of the wild type from 2 DPA to 20 DPA (Figure 2I–K), suggesting a lower elongation rate in the knockout fibers. We also observed that fast fiber elongation ceased at 15 DPA in the *GhMYB201* knockout line, 3 days earlier than that in the wild type (18 DPA) (Figure 2I–K). Consistently, the birefringence of fiber walls was observed in the *GhMYB201* knockout line at 13 DPA (Figure S3), indicating an earlier onset of secondary wall deposition in the knockout fibers. Therefore, the knockout of *GhMYB201* led to impaired fiber elongation and final length by decreasing the rate and also duration of fiber elongation.

### 2.3. GhMYB201 Transcriptionally Activates Cell Wall Loosening-Related Genes

**Figure 3:**
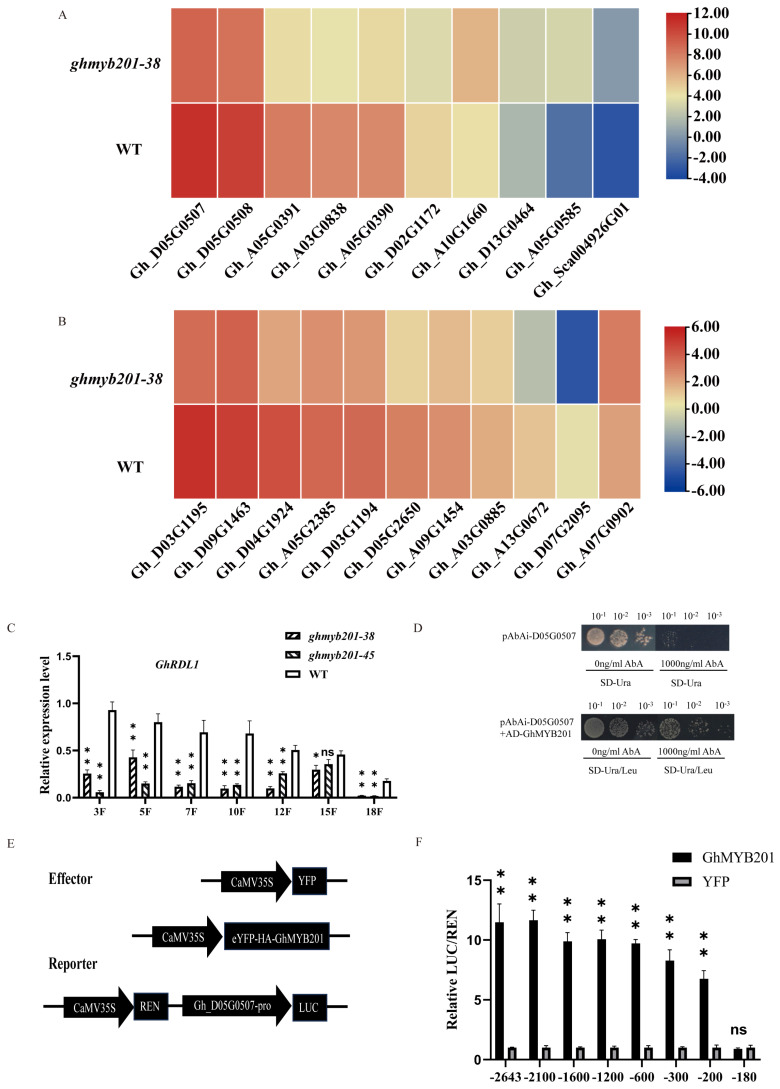
*GhRDL1* is the direct target of GhMYB201. (**A**) Expression heatmap of *GhRDL* genes. (**B**) Expression heatmap of *Expansin* genes. (**C**) qRT-PCR of *GhRDL1* in the cotton fiber of knockout lines and WT. Data are presented as the means ± SD. * *p* < 0.05, ** *p* < 0.01, ns means no statistically significant difference (Student’s *t*-test). (**D**) Yeast-one-hybridization used the promoter of *GhRDL1* and GhMYB201. (**E**) Schematic structures of the effector and reporter used for transient expression analysis. 35S-YFP-HA-GhMYB201 and 35S-YFP were used as effectors, and LUC driven by *GhRDL1* promoters were used as reporters. (**F**) Transactivation assay of a series of deletions of the *GhRDL1* promoter revealed that the DNA fragment located between −180 and −200 bp upstream of the start codon was sufficient for GhMYB201 activation. Data are presented as the means ± SD. ** *p* < 0.01, ns means no statistically significant difference (Student’s *t*-test).

To identify GhMYB201-regulated genes in elongating fibers, we performed transcriptome sequencing (RNA-seq) analysis of 7 DPA fibers from the wild type and *GhMYB201* knockout line (*ghmyb201-38*). A total of 5762 differentially expressed genes (DEGs), including 1674 downregulated and 4088 upregulated genes, were identified in knockout elongation fibers (Figure S5). In the significantly downregulated DEGs (i.e., GhMYB201 activated genes in wild type fibers), we recognized a series of cell wall loosening-related genes (Figure 3A,B), especially BURP domain protein (RDL, AtRD22-Like) and expansin genes [11].

Further, *GhRDL1* (*Gh_D05G0507*) with a high expression level in elongating fibers was selected to analyze whether it could be transcriptionally activated by GhMYB201. qRT-PCR analysis showed that *GhRDL1* transcript levels in elongating fibers of 3-18 DPA were significantly decreased in *GhMYB201* knockout lines compared with WT (Figure 3C). In the yeast one-hybrid (Y1H) assay, yeast cells harbored with pGADT7-GhMYB201 and pAbAi containing promoter regions of *GhRDL1* survived on selective medium containing aureobasidin A (AbA; 1000 ng/mL), suggesting that GhMYB201 interacted with the promoter of *GhRDL1* in yeast (Figure 3D). A dual-luciferase fluorescence assay was performed to detect the transcriptional activation activity of GhMYB201 on the *GhRDL1* promoters of various lengths. As shown in Figure 3E,F, GhMYB201 could bind the *GhRDL1* promoter and activate the expression of the downstream reporter gene (firefly luciferase, LUC), and further, the binding site was located in a 20 bp fragment (−200 bp and −180 bp upstream of ATG). These results collectively demonstrated that GhMYB201 activated the expression of cell wall loosening genes in elongating cotton fibers.

### 2.4. GhMYB201 Activates the Expression of GhKCSs and Changes the VLCFA Contents

**Figure 4:**
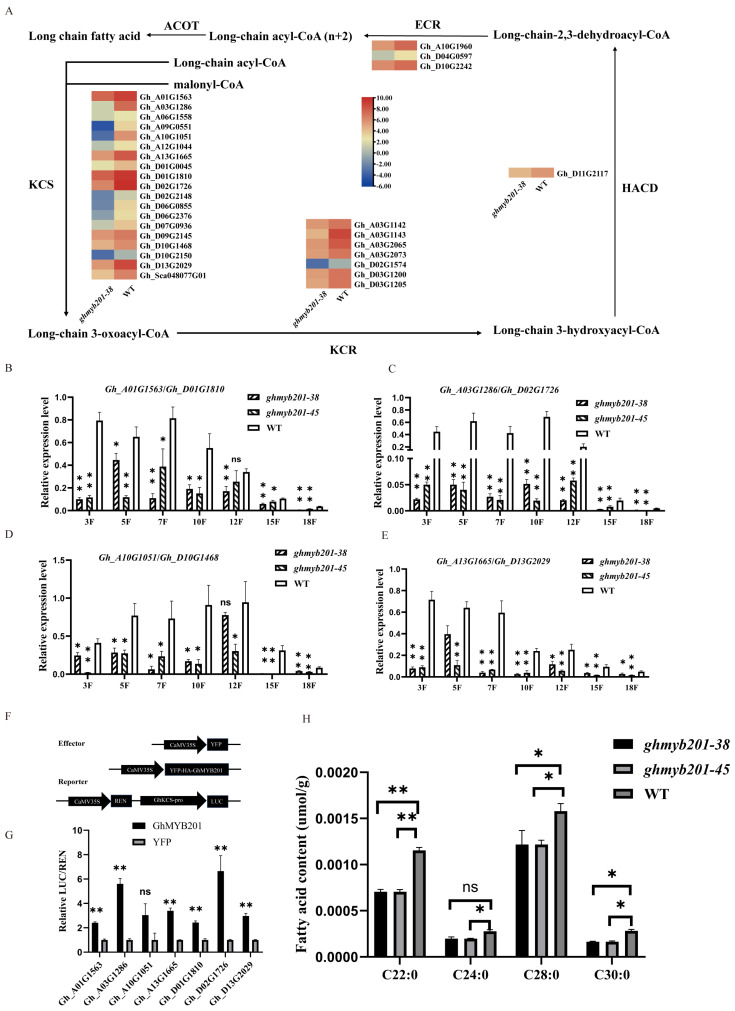
GhMYB201 enhances VLCFA biosynthesis and the expression of synthase genes. (**A**) Heatmap of fatty acid elongation pathway and related gene expression. Heatmap showing genes significantly differentially expressed between *ghmyb201-38* and WT. Different colors represent log_2_(Fold change). (**B**–**E**) qRT-PCR of *GhKCSs* in the cotton fiber of knockout lines and WT. Data are presented as the means ± SD. * *p* < 0.05, ** *p* < 0.01, ns means no statistically significant difference (Student’s *t*-test). (**F**) Schematic structures of the effector and reporter used for transient expression analysis. (**G**) Effects of GhMYB201 on the activity of GhKCSs. 35S-YFP-HA-GhMYB201 and 35S-YFP were used as effectors, and LUC driven by GhKCSs promoters were used as reporters. Data are presented as the means ± SD. ** *p* < 0.01, ns means no statistically significant difference (Student’s *t*-test). (**H**) Fatty acid contents of *ghmyb201* knockout lines and wild type fibers at 10 DPA. Data are presented as the means ± SD. * *p* < 0.05, ** *p* < 0.01, ns means no statistically significant difference (Student’s *t*-test).

KEGG and GO enrichment analyses of the downregulated DEGs showed that multiple VLCFA-related processes, including fatty acid elongation, fatty acid biosynthesis, biosynthesis of unsaturated fatty acid degradation, and fatty acid metabolic process, were significantly enriched (Figure S4B,C). VLCFAs, synthesized via the fatty acid elongation pathway, are important components that promote cotton fiber elongation [9,12,14]. Further analysis indicated that four out of five enzymes in the fatty acid elongation pathway, namely 3-ketoacyl-CoA synthase (KCS), very-long-chain 3-oxoacyl-CoA reductase (KCR), very-long-chain (3R)-3-hydroxyacyl-CoA dehydratase (HACD), and very-long-chain enoyl-CoA reductase (ECR), were downregulated in the *GhMYB201* knockout line compared with the wild type (Figure 4A). qRT-PCR analyses consistently indicated that the expression level of the 8 *GhKCSs* in elongating fibers of 3–18 DPA was significantly decreased in knockout lines compared with the wild type (Figure 4B–E). Furthermore, the contents of saturated VLCFAs (C22:0, C24:0, C28:0, and C30:0) in *GhMYB201* knockout 10 DPA fibers were significantly lower than those of WT (Figure 4H). Finally, the dual-luciferase assay showed that GhMYB201 could strongly activate the expression of the downstream firefly luciferase gene (Figure 4F,G), suggesting that GhMYB201 could bind to the promoter of *GhKCSs* and activate their transcription. These data indicated that GhMYB201 promoted VLCFA biosynthesis in elongating fibers via activating VLCFA synthase genes.

**Figure 5:**
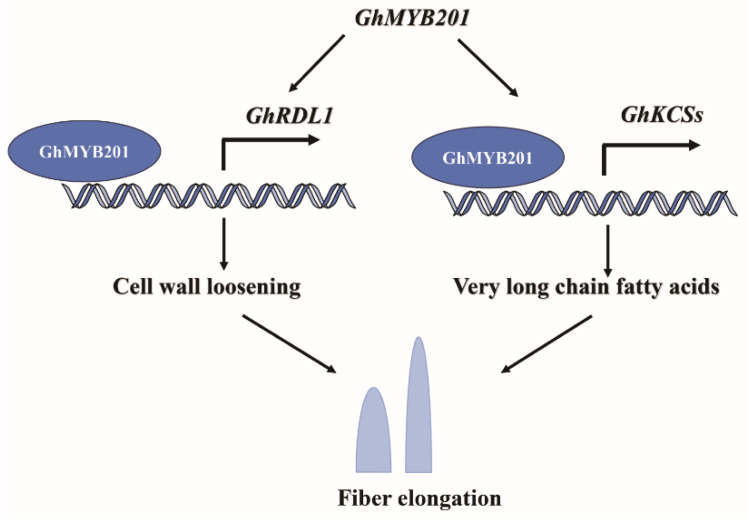
The molecular model that GhMYB201 promotes fiber elongation. Proposed working model of the mechanism by which *GhMYB201* regulates fiber elongation. *GhMYB201* is preferentially expressed during fiber elongation. GhMYB201 protein directly activated the expression of GhRDL1 (cell wall loosening protein) and GhKCSs (β-ketoacyl-CoA synthase) to promote fiber elongation.

Taken together, our results support the function of GhMYB201 in fiber elongation. GhMYB201 promotes cell wall loosening via activating the expression of *GhRDLs* and increases the VLCFA levels by upregulating the β-ketoacyl-CoA synthase genes (*GhKCSs*), which results in elongated fibers (Figure 5). Our results provide new insight into the molecular mechanism regulating cotton fiber elongation by revealing the function of GhMYB201 in cotton fiber development, contributing to improving fiber quality through GhMYB201 gene manipulation.

## 3. Discussion

Transcription factors play essential roles in regulating the elongation or primary cell wall synthesis of cotton fibers. A series of transcription factors have been reported to promote fiber elongation via activating structural genes and/or responding to various stimuli, including phytohormone signals. Nevertheless, a lot of transcription factors still need to be functionally dissected to understand the regulatory mechanism of fiber elongation comprehensively. In this study, an R2R3-MYB transcription factor gene *GhMYB201*, preferentially expressed in elongating fibers, was characterized and functionally verified via CRISPR/Cas9-mediated gene editing. GhMYB201s was homologous to *Arabidopsis* AtMYB60 and characterized as a typical transcription factor with nuclear location in tobacco and transactivation activity in yeast (Figure 1D,E). Knocking out of *GhMYB201s* significantly reduced the rate and duration of fiber elongating and the final length of mature fibers. Furthermore, we demonstrated that GhMYB201 can bind to the promoters and activate the transcription of cell wall loosening genes (e.g., *GhRDLs*) and VLCFA synthase genes (i.e., *GhKCSs*), thereby enhancing VLCFA levels in elongating fibers. Taken together, we revealed the functions and possible mechanism of a new transcription factor GhMYB201 in promoting fiber elongation, which added new clues to establish a complete regulatory network of cotton fiber elongation.

AtMYB60 was first identified as an R2R3-MYB transcription factor regulating stomatal movement and drought tolerance, which is expressed exclusively in guard cells of all epidermal tissues in *Arabidopsis* [26]. It was further indicated that AtMYB60 plays dual roles under drought stress by controlling stomatal movement and root growth. At the initial stage of drought stress, *AtMYB60* expression is induced by low-level ABA to enhance root growth for increased water uptake, while severe drought stress inhibits the expression of the *AtMYB60* gene, resulting in stomatal closure and root growth inhibition [27]. In addition, overexpression of *AtMYB60* represses anthocyanin biosynthesis in lettuce leaves [29]. In cotton, three and five to six AtMYB60 homologs were identified in diploid and tetraploid species (Figure 1C and Figure S1), respectively. Previously, Xu et al. [23] reported that drought-induced *GbMYB60* (homologous to *GhMYB192*, *Ghi_A13G07006/Ghi_D13G05886*) was expressed in the vascular tissue and meristems, and its overexpression negatively regulates salt tolerance in *Arabidopsis*. GhMYB201 shared high sequence similarity with AtMYB60 (Figure 1A), and the overexpression of *GhMYB201* promoted stomatal opening in *Arabidopsis* (Figure S4), suggesting the similar function of *GhMYB201* as *AtMYB60* to regulate stomatal movement. However, *GhMYB201* was specifically expressed in the elongating fiber stage, and knockout of *GhMYB201* led to significantly shorter cotton fibers (Figure 2D), indicating that GhMYB201 functioned as a positive regulator of the rapid elongation of fiber cells. Taken together, in addition to stomatal movement, the AtMYB60 family might be involved in regulating multiple physiological processes, including root growth, secondary metabolism, and cell elongation.

Cotton fiber is one of the longest plant cells and is regarded as an excellent model to explore cell growth [3,30]. Cotton fiber cells elongate through a combination of tip growth and diffuse-growth modes, which require repeated cell wall loosening and integration of new components into the wall. Cell wall loosening proteins, including BURP domain protein (e.g., GhRDL1), expansin (e.g., GhEXPA1), and xyloglucan endotransglycosylase/hydrolase, play important roles in promoting cotton fiber elongation [11,31]. Both *GhRDL1* and *GhEXPA1* are direct targets of the fiber elongation-promoting transcription factor GhHOX3. Meanwhile, GA repressor GhSLR1 interacts with GhHOX3 to inhibit its transcriptional activation activity on *GhRDL1* and *GhEXPA1* [15]. Therefore, cell wall loosening mediated by GhRDL1 and GhEXPA1 is a part of GA signaling to promote fiber cell elongation. In this study, we demonstrated that GhMYB201 can directly bind to the promoter and activate the expression of *GhRDL1* and possibly other *GhRDLs* and *GhEXPAs*, suggesting that GhMYB201 promoted fiber elongation via GhRDLs- and GhEXPAs-mediated cell wall loosening. Considering that GhMYB201 did not interact with GhHOX3, the GhMYB201 regulation of cell wall loosening and fiber elongation might be independent of GA signaling. Furthermore, we found that GhMYB201 might transcriptionally activate more efficiently than GhHOX3 (Figure S6), indicating the importance of the GhMYB201 pathway to regulate fiber elongation.

Numerous studies reported that saturated very-long-chain fatty acids (VLCFAs) are involved in promoting fiber elongation [9,12,13,14,32]. Overexpression of the key VLCFA synthase gene *GhKCS10_At* (*GhKCS6*) significantly increases fiber length, while suppression of *GhKCS10_At* leads to a decrease in fiber length [14,32]. VLCFAs activate ACC oxidase genes (*GhACO*s) expression and ethylene biosynthesis [12]. Meanwhile, its synthase genes (especially *GhKCS*s) are upregulated in response to GA, BR, and SL signals [9,13,14]. Biochemical and RNA-Seq analyses revealed that VLCFA biosynthesis pathways were significantly decreased in *ghmyb201* knockout fibers (Figure 4A–G). These observations indicated that VLCFA biosynthesis genes might be the direct target of GhMYB201. Besides as signaling molecules, VLCFAs were also precursors of sphingolipids, seed triacylglycerols, suberins, and cuticular waxes [3]. The mature fibers of GhMYB201 knockout cotton cohered to each other and the seed (Figure 1D) with a coarse appearance, in contrast to the smooth appearance of the wild type fibers (Figure 2H). We envision that this phenotypic variation might be attributed to disturbed cuticular wax biosynthesis due to insufficient VLCFA precursors in elongating fibers, although more detailed work is still ongoing.

## 4. Materials and Methods

### 4.1. Identification and Cloning of GhMYB201s

Using the previously published transcriptomic data [22] of 20 tissues (fibers of 5, 10, 20, and 25 DPA, ovules of −3, −1, 0, 1, 3, 5, 10, 20, 25, and 35 DPA, roots, stems, leaves, petals stamens, and pistils), we calculated the enrichment fold and the enrichment factors of all genes. The enrichment fold was calculated as the average FPKM (Fragments Per Kilobase of exon model per Million mapped fragments) in elongating fiber of 5 and 10 DPA/average FPKM in all tissues, and the enrichment factor as the enrichment fold multiply the average FPKM of elongating fibers. The transcription factor genes preferentially expressed in elongating fibers were identified with the cutoff (enrichment fold > 3 and enrichment factor > 50, Table S1). *GhMYB201*s (*GhMYB201Dt* and *GhMYB201At*) own the highest enrichment fold and enrichment factor.

The 5 DPA fiber cDNA was used as the template to amplify *Gh_D13G1712* coding sequences with PrimeSTAR^®^Max DNA Polymerase (TaKaRa, Dalian, China). The coding sequences of Gh_D13G1712 were cloned into a pLGN vector linearized with *Eco*RI and *Bcu*I to construct the pro35S-GhMYB201 vector using the ClonExpress II One Step Cloning Kit (Vazyme, Nanjing, China). The cloned fragment was confirmed by Sanger sequencing in Tsingke (Beijing, China). Primers used in this assay are listed in Supplementary Table S3.

### 4.2. Total RNA Isolation, qRT-PCR Analysis, and Transcriptome Analysis

Total RNAs were extracted from various cotton tissues using the RNA Easy Fast Plant Tissue Kit (Tiangen, Beijing, China) according to the instructions provided by the manufacturer. First-strand cDNA was reverse-transcribed using a PrimeScriptTM RT reagent kit (TaKaRa, Dalian, China) with a gDNA eraser. Quantitative PCRs (qRT-PCR) were performed with SYBR-Green PCR MasterMix (Vazyme, Nanjing, China). qRT-PCR assays were performed using a CFX96 real-time PCR system (Bio-Rad, CA, United States). The parameters of the qRT-PCR assay were as follows: 95 °C for 1 min, followed by 40 cycles of 95 °C for 10 s and 60 °C for 30 s. Specific primers of *GhMYB201*, *GhRDL1*, and *GhKCSs* were designed. Cotton GhUBQ14 and GhActin2 were used as internal controls to normalize the transcript levels of target genes. Primers used in this assay are listed in Supplementary Table S3.

Total RNAs were extracted from 7 DPA fibers of knockout line #38 and the wild type and were detected and sequenced by Shanghai Majorbio Bio-pharm Technology Co., Ltd. (www.majorbio.com (accessed on 1 February 2021)). After filtration, paired-end clean reads were assembled to the genome assembly of *G. hirsutum* (https://mascotton.njau.edu.cn/Data.htm (accessed on 3 March 2020)) using HISAT2 [33,34]. The number of fragments per kilobase per million mapped reads (FPKM) was used to normalize and calculate the expression level of each gene. Differentially expressed genes (DEGs) were identified with the cutoffs: |log2(fold change)| ≥ 1 and FDR (false discovery rate) < 0.05. The Gene Ontology (GO) and Kyoto Encyclopedia of Genes and Genomes (KEGG) analyses were performed using topGO and KOBAS 3.0, respectively [35,36].

### 4.3. Generation of Knockout Cottons

The specific guide RNA (sgRNA) sequences targeting *GhMYB201Dt* and *GhMYB201At* were designed on the website (http://crispr.hzau.edu.cn/CRISPR/ (accessed on 2 January 2020)) [37]. Two sgRNA sequences (5′CCTTAGCTTCTTCTTCAGAT3′ and 5′TATGGAGCCTCCTTCAATGG3′) and tRNA fusion were amplified through PCR using the pUC-sgRNA-tRNA vector as a template and then cloned into pRGEB32-GhU6.9 expression vector digested with the *Eco*31I [38]. The pRGEB-*ghmyb201* construct was transferred into *Agrobacterium tumefaciens* strain LBA4404. The Agrobacterium-mediated transformation was performed according to the previously described method [39]. Cotton plants (*G. hirsutum* cv Jimian14 and transgenic *ghmyb201* knockout lines) were grown in the greenhouse at Southwest University, Chongqing, China. On the day of anthesis, the flowers and bolls were marked as 0 DPA. Primers used in this assay are listed in Supplementary Table S3.

### 4.4. Observation of Cotton Fiber Phenotype

Cotton bolls of *ghmyb201* knockout lines and wild type on similar fruit branches under the same growing conditions were harvested. The fiber length was combed and measured manually. The 6, 8, 10, 12, 15, 18, and 20 DPA cottons were harvested. The ovules with fibers were boiled in 30% acetic acid. Then, the fiber length was measured manually. The fresh ovules and mature fibers of transgenic plants and wild type plants were collected and observed using a scanning electron microscope (SEM) (SU 3500, Hitachi, Tokyo, Japan). Cross-sections of mature fibers were performed and observed as described [40]. The cell wall thickness of fiber transverse sections was measured by ImageJ (https://imagej.net/software/fiji/ (accessed on 4 October 2022)).

About 15 g of mature fibers for each sample were collected. The mature fibers were tested by the Center of Cotton Fiber Quality Inspection and Testing, Ministry of Agriculture and Rural Affairs (Henan, China). Fiber length, strength, micronaire, and uniformity were measured with a high-volume fiber test system (Premier HFT 9000, Coimbatore, India).

### 4.5. Arabidopsis Growth and Transformation

*Arabidopsis thaliana*, ecotype Columbia (Col-0) was used in this study. The mutant line (SALK_148646C) was obtained from the *Arabidopsis* Biological Resource Center (ABRC) (https://abrc.osu.edu/ (accessed on 3 March 2020)). Seeds were surface sterilized with ethanol (75%), followed by washing 3 times with sterile water. *Arabidopsis* seeds were germinated and grown at 22 °C with a 16 h light/8 h dark cycle at a relative humidity of 70% following 2 days of stratification at 4 °C. The plants were transferred to soil and grown in a greenhouse after 2 weeks. The pro35S-GhMYB201 construct was introduced into *A. tumefaciens* strain GV3101 by electroporation (MicroPulser, Bio-Rad, Hercules, CA, USA). The overexpression of *GhMYB201* in *Arabidopsis* was transformed using floral dip [41]. Transformed seeds were determined by PCR using the specific primers. The expression level of GhMYB201 was examined by qRT-PCR using the rosette leaf cDNA as a template. *Arabidopsis* AtActin2 was used as an internal control to normalize the transcript levels of target genes. Primers used in this assay are listed in Supplementary Table S3.

Leaves of 5-week-old seedlings were used in the stomatal aperture assays. Fully expanded leaves were detached and submerged in an opening solution (5 mM KCl, 50 mM CaCl_2_, and 10 mM MES buffer, pH 5.6) for 2 h. Leaf peels were prepared and observed with an optical microscope (Olympus IX81, Olympus, Tokyo, Japan).

### 4.6. Transactivation Activity Assay in Yeast and Yeast One-Hybrid Assay

To investigate the transcriptional activity of GhMYB201, the full-length or truncated coding sequence (CDS) of GhMYB201 was amplified and inserted into the pGBKT7 with *EcoR*I and *BamH*I restriction sites using the ClonExpress II One Step Cloning Kit (Vazyme, Nanjing, China). The pGBKT7 bait vector was transferred into yeast strain Y2H using the high-efficiency lithium acetate transformation and plated on a minimal synthetic-defined (SD) base supplemented with -Trp medium for 3 days at 30 °C. The transcriptional activity was detected on SD/-Trp-His-Ade medium with X-α-Gal. Primers used in this assay are listed in Supplementary Table S3.

Yeast one-hybrid (Y1H) assay was performed with the Match-maker™ Gold Yeast One-Hybrid System (Clontech). The promoter fragments of *Gh_D05G0507* were amplified and cloned into the pAbAi vectors with *Hind*III and *Xho*I restriction sites. The pAbAi vector was linearized with *BstBI*, then transformed into Y1H Gold strain to generate a specific reporter strain and plated on SD/-Ura media supplemented with appropriate concentrations of Aureobasidin A (AbA). The full-length CDS sequences of *GhMYB201* were cloned into the modified pGADT7 vector digested with *EcoR*I and *Bcu*I. The plasmid was transformed into Y1H Gold strains containing *Gh_D05G0507*-pAbAi and plated on SD/-Leu-Ura media supplemented with AbA. All strains were cultured at 30 °C for 2 to 3 days. Primers used in this assay are listed in Supplementary Table S3.

### 4.7. Transient Assays in Nicotiana Benthamiana

*N. benthamiana* plants were grown in the growth chamber at 23 °C and 16 h light/8 h dark cycles. The *YFP* and *YFP-HA-GhMYB201* fusion genes were amplified and cloned into a pLGN vector linearized with *EcoR*I and *Bcu*I. The pro35S-YFP-HA-GhMYB201 plasmid and pro35S-YFP plasmid were introduced into *A. tumefaciens* strain GV3101 by electroporation (MicroPulser, Bio-Rad, CA, USA). The transformed Agrobacterium colony containing pro35S-YFP-HA-GhMYB201/pro35S-YFP was grown overnight at 28 °C in an antibiotic selection medium containing rifampicin and kanamycin 50 mg/L. The cells were collected (5000 rpm, 10 min) when cultured to OD_600_ of 0.8–1.0, and then resuspended in infiltration solution (10 mM MgCl_2_, 10mM MES, and 100 μM acetosyringone). The resuspended cells were injected into 5-week-old *N. benthamiana* leaves. Two days later, the leaves were stained by 4,6-diamino-2-phenyl indole (DAPI, 5 μg/mL) for 10 min and then washed with ddH_2_O 3 times. YFP fluorescence signal was excited at 514 nm by laser confocal microscope (SP8, Leica, Wetzlar, Germany). Primers used in this assay are listed in Supplementary Table S3.

About 2000bp of *GhRDL1* and *GhKCSs* promoters were amplified from *G. hirsutum* cv Jimian14 gDNA and inserted into the pGreen0800 vector with *Nco*I and *Kpn*I restriction enzyme sites. The successfully constructed vectors were transferred into *A. tumefaciens* strain GV3101 along with pSOUP vector. The pro35S-YFP-HA-GhMYB201 or pro35S-YFP were used as the effectors. The Agrobacteria harboring reporter and effectors were coin-filtrated into 5-week-old *N. benthamiana* leaves, as described above. The leaf discs at the infiltrated areas were collected two days later and ground into powder in liquid nitrogen. The measurement of LUC activity was detected using a dual-luciferase assay system (Vazyme, Nanjing, China). Primers used in this assay are listed in Supplementary Table S3.

### 4.8. Fatty Acid Extractions

Ten DPA fibers of *ghmyb201* knockout lines and wild type were collected and ground into powder in liquid nitrogen. The 100 mg samples were inactivated with hot isopropanol (75 °C) using a protocol previously described [42]. Following inactivation, 1.2 mL of Extraction solvent containing chloroform: methanol: 300 mM ammonium acetate (30:41.5:3.5) (*v*/*v*/*v*) was added to the samples followed by incubating at room temperature for 24 h at 150 rpm. After incubation, samples were centrifuged (12,000× *g*, 10 min), and clear supernatant was transferred to fresh tubes. The inactivation and extraction steps were repeated once and lipid extracts from both rounds of extraction were pooled and dried in a SpeedVac (Genevac, Ipswich, UK). The dried extract was resuspended in 150 μL methanol and derivatized using 50 mM 3-Nitrophenylhdyrazine [43]. Metabolites were analyzed on a Jasper HPLC coupled to the Sciex 4500 MD system. In brief, individual metabolites were separated on a Phenomenex Kinetex C18 column (100 × 2.1 mm, 2.6 μm) using 0.05% formic acid in acetonitrile: water (1:9) as mobile phase A and 0.05% formic acid and 2 mM ammonium acetate in acetonitrile: methanol: isopropanol (1:2:2) as mobile phase B. VLCFAs were quantitated using d31-16:0 (Sigma-Aldrich, St. Louis, MO, USA) and d8-20:4 (Cayman Chemicals, Ann Arbor, MI, USA) as internal standards.

## 5. Conclusions

In this study, we characterized an R2R3 transcription factor GhMYB201, which was localized in the nucleus. qRT-PCR analysis revealed that *GhMYB201* was dominantly expressed in rapid elongation fibers. Knockout of *ghmyb201* resulted in shorter fibers compared with the wild type due to decreased expression of cell wall loosening genes (*GhRDLs*) and β-ketoacyl-CoA synthase genes (*GhKCSs*). Our findings suggest that GhMYB201 is crucial for promoting fiber elongation, providing a new genetic strategy for improving fiber quality.

## Figures and Tables

**Table 1:** Fiber quality index of GhMYB201 knockout cotton lines (*ghmyb201-38* and *-45*) and wild-type (WT) controls.

	Average Length of Upper Quartile Fibers (mm)	Length Uniformity (%)	Fiber Strength (cN/tex)	Micronaire Value	Fiber Elongation (%)
WT	29.5 ± 0.2	84.65 ± 0.95	31.6 ± 0.80	5.15 ± 0.05	6.40 ± 0.40
*ghmyb201-38*	21.25 ± 0.35 **	79.5 ± 0.20 *	23.45 ± 1.15 *	7.1 ± 0.10 **	6.10 ± 0.20 ^ns^
*ghmyb201-45*	22.25 ± 0.35 **	81.45 ± 0.05 ^ns^	23.5 ± 0.30 *	7.25 ± 0.25 *	6.40 ± 0.10 ^ns^

Note: Data are presented as the means ± SD. * *p* < 0.05, ** *p* < 0.01, ^ns^ means no statistically significant difference (Student’s *t*-test).

## Data Availability

The data presented in this study are available on request from the corresponding authors due to privacy.

## Supplementary Materials

The following supporting information can be downloaded at: https://www.mdpi.com/article/10.3390/ijms25179559/s1.

## References

[1] Haigler C.H., Betancur L., Stiff M.R., Tuttle J.R. (2012). Cotton fiber: A powerful single-cell model for cell wall and cellulose research. Front. Plant Sci..

[2] Lee J.J., Woodward A.W., Chen Z.J. (2007). Gene expression changes and early events in cotton fibre development. Ann. Bot..

[3] Qin Y.M., Zhu Y.X. (2011). How cotton fibers elongate: A tale of linear cell-growth mode. Curr. Opin. Plant Biol..

[4] Wang N.N., Li Y., Chen Y.H., Lu R., Zhou L., Wang Y., Zheng Y., Li X.B. (2021). Phosphorylation of WRKY16 by MPK3-1 is essential for its transcriptional activity during fiber initiation and elongation in cotton (*Gossypium hirsutum*). Plant Cell.

[5] Cao J.F., Zhao B., Huang C.C., Chen Z.W., Zhao T., Liu H.R., Hu G.J., Shangguan X.X., Shan C.M., Wang L.J. (2020). The miR319-Targeted GhTCP4 Promotes the Transition from Cell Elongation to Wall Thickening in Cotton Fiber. Mol. Plant.

[6] Ding X., Li X., Wang L., Zeng J., Huang L., Xiong L., Song S., Zhao J., Hou L., Wang F. (2021). Sucrose enhanced reactive oxygen species generation promotes cotton fibre initiation and secondary cell wall deposition. Plant Biotechnol. J..

[7] Wen X., Chen Z., Yang Z., Wang M., Jin S., Wang G., Zhang L., Wang L., Li J., Saeed S. (2023). A comprehensive overview of cotton genomics, biotechnology and molecular biological studies. Sci. China Life Sci..

[8] Wang N.N., Ni P., Wei Y.L., Hu R., Li Y., Li X.B., Zheng Y. (2024). Phosphatidic acid interacts with an HD-ZIP transcription factor GhHOX4 to influence its function in fiber elongation of cotton (*Gossypium hirsutum*). Plant J..

[9] He P., Zhu L., Zhou X., Fu X., Zhang Y., Zhao P., Jiang B., Wang H., Xiao G. (2024). Gibberellic acid promotes single-celled fiber elongation through the activation of two signaling cascades in cotton. Dev. Cell.

[10] Wang Y., Li Y., He S.P., Xu S.W., Li L., Zheng Y., Li X.B. (2023). The transcription factor ERF108 interacts with AUXIN RESPONSE FACTORs to mediate cotton fiber secondary cell wall biosynthesis. Plant Cell.

[11] Xu B., Gou J.Y., Li F.G., Shangguan X.X., Zhao B., Yang C.Q., Wang L.J., Yuan S., Liu C.J., Chen X.Y. (2013). A cotton BURP domain protein interacts with alpha-expansin and their co-expression promotes plant growth and fruit production. Mol. Plant.

[12] Qin Y.M., Hu C.Y., Pang Y., Kastaniotis A.J., Hiltunen J.K., Zhu Y.X. (2007). Saturated very-long-chain fatty acids promote cotton fiber and *Arabidopsis* cell elongation by activating ethylene biosynthesis. Plant Cell.

[13] Tian Z., Zhang Y., Zhu L., Jiang B., Wang H., Gao R., Friml J., Xiao G. (2022). Strigolactones act downstream of gibberellins to regulate fiber cell elongation and cell wall thickness in cotton (*Gossypium hirsutum*). Plant Cell.

[14] Yang Z., Liu Z., Ge X., Lu L., Qin W., Qanmber G., Liu L., Wang Z., Li F. (2023). Brassinosteroids regulate cotton fiber elongation by modulating very-long-chain fatty acid biosynthesis. Plant Cell.

[15] Shan C.M., Shangguan X.X., Zhao B., Zhang X.F., Chao L.M., Yang C.Q., Wang L.J., Zhu H.Y., Zeng Y.D., Guo W.Z. (2014). Control of cotton fibre elongation by a homeodomain transcription factor GhHOX3. Nat. Commun..

[16] Zhao B., Cao J.F., Hu G.J., Chen Z.W., Wang L.Y., Shangguan X.X., Wang L.J., Mao Y.B., Zhang T.Z., Wendel J.F. (2018). Core cis-element variation confers subgenome-biased expression of a transcription factor that functions in cotton fiber elongation. New Phytol..

[17] Zeng J., Yao D., Luo M., Ding L., Wang Y., Yan X., Ye S.E., Wang C., Wu Y., Zhang J. (2023). Fiber-specific increase of carotenoid content promotes cotton fiber elongation by increasing abscisic acid and ethylene biosynthesis. Crop J..

[18] Machado A., Wu Y., Yang Y., Llewellyn D.J., Dennis E.S. (2009). The MYB transcription factor GhMYB25 regulates early fibre and trichome development. Plant J..

[19] Pu L., Li Q., Fan X., Yang W., Xue Y. (2008). The R2R3 MYB transcription factor GhMYB109 is required for cotton fiber development. Genetics.

[20] Sun W., Gao Z., Wang J., Huang Y., Chen Y., Li J., Lv M., Wang J., Luo M., Zuo K. (2019). Cotton fiber elongation requires the transcription factor GhMYB212 to regulate sucrose transportation into expanding fibers. New Phytol..

[21] Xu F., Li G., He S., Zeng Z., Wang Q., Zhang H., Yan X., Hu Y., Tian H., Luo M. (2024). Sphingolipid inhibitor response gene GhMYB86 controls fiber elongation by regulating microtubule arrangement. J. Integr. Plant Biol..

[22] Zhang T., Hu Y., Jiang W., Fang L., Guan X., Chen J., Zhang J., Saski C.A., Scheffler B.E., Stelly D.M. (2015). Sequencing of allotetraploid cotton (*Gossypium hirsutum* L. acc. TM-1) provides a resource for fiber improvement. Nat. Biotechnol..

[23] Xu F.C., Liu H.L., Xu Y.Y., Zhao J.R., Guo Y.W., Long L., Gao W., Song C.P. (2018). Heterogeneous expression of the cotton R2R3-MYB transcription factor GbMYB60 increases salt sensitivity in transgenic. Plant Cell Tissue Org..

[24] Walford S.A., Wu Y., Llewellyn D.J., Dennis E.S. (2011). GhMYB25-like: A key factor in early cotton fibre development. Plant J..

[25] Wu A., Lian B., Hao P., Fu X., Zhang M., Lu J., Ma L., Yu S., Wei H., Wang H. (2024). GhMYB30-GhMUR3 affects fiber elongation and secondary wall thickening in cotton. Plant J..

[26] Cominelli E., Galbiati M., Vavasseur A., Conti L., Sala T., Vuylsteke M., Leonhardt N., Dellaporta S.L., Tonelli C. (2005). A guard-cell-specific MYB transcription factor regulates stomatal movements and plant drought tolerance. Curr. Biol..

[27] Oh J.E., Kwon Y., Kim J.H., Noh H., Hong S.W., Lee H. (2011). A dual role for MYB60 in stomatal regulation and root growth of *Arabidopsis thaliana* under drought stress. Plant Mol. Biol..

[28] Wang C., Lv Y., Xu W., Zhang T., Guo W. (2014). Aberrant phenotype and transcriptome expression during fiber cell wall thickening caused by the mutation of the Im gene in immature fiber (im) mutant in *Gossypium hirsutum* L.. BMC Genom..

[29] Park J.S., Kim J.B., Cho K.J., Cheon C.I., Sung M.K., Choung M.G., Roh K.H. (2008). *Arabidopsis* R2R3-MYB transcription factor AtMYB60 functions as a transcriptional repressor of anthocyanin biosynthesis in lettuce (*Lactuca sativa*). Plant Cell Rep..

[30] Kim H.J., Triplett B.A. (2001). Cotton Fiber Growth in Planta and in Vitro. Models for Plant Cell Elongation and Cell Wall Biogenesis. Plant Physiol..

[31] Lee J., Burns T.H., Light G., Sun Y., Fokar M., Kasukabe Y., Fujisawa K., Maekawa Y., Allen R.D. (2010). Xyloglucan endotransglycosylase/hydrolase genes in cotton and their role in fiber elongation. Planta.

[32] Huang G., Wu Z., Percy R.G., Bai M., Li Y., Frelichowski J.E., Hu J., Wang K., Yu J.Z., Zhu Y. (2020). Genome sequence of Gossypium herbaceum and genome updates of *Gossypium arboreum* and *Gossypium hirsutum* provide insights into cotton A-genome evolution. Nat. Genet..

[33] Fang L., Wang Q., Hu Y., Jia Y., Chen J., Liu B., Zhang Z., Guan X., Chen S., Zhou B. (2017). Genomic analyses in cotton identify signatures of selection and loci associated with fiber quality and yield traits. Nat. Genet..

[34] Kim D., Langmead B., Salzberg S.L. (2015). HISAT: A fast spliced aligner with low memory requirements. Nat. Methods.

[35] Aibar S., Fontanillo C., Droste C., De Las Rivas J. (2015). Functional Gene Networks: R/Bioc package to generate and analyse gene networks derived from functional enrichment and clustering. Bioinformatics.

[36] Bu D., Luo H., Huo P., Wang Z., Zhang S., He Z., Wu Y., Zhao L., Liu J., Guo J. (2021). KOBAS-i: Intelligent prioritization and exploratory visualization of biological functions for gene enrichment analysis. Nucleic Acids Res..

[37] Lei Y., Lu L., Liu H.Y., Li S., Xing F., Chen L.L. (2014). CRISPR-P: A web tool for synthetic single-guide RNA design of CRISPR-system in plants. Mol. Plant.

[38] Wang P., Zhang J., Sun L., Ma Y., Xu J., Liang S., Deng J., Tan J., Zhang Q., Tu L. (2018). High efficient multisites genome editing in allotetraploid cotton (*Gossypium hirsutum*) using CRISPR/Cas9 system. Plant Biotechnol. J..

[39] Luo M., Xiao Y., Li X., Lu X., Deng W., Li D., Hou L., Hu M., Li Y., Pei Y. (2007). GhDET2, a steroid 5alpha-reductase, plays an important role in cotton fiber cell initiation and elongation. Plant J..

[40] Yan Q., Wang Y., Li Q., Zhang Z., Ding H., Zhang Y., Liu H., Luo M., Liu D., Song W. (2018). Up-regulation of GhTT2-3A in cotton fibres during secondary wall thickening results in brown fibres with improved quality. Plant Biotechnol. J..

[41] Clough S.J., Bent A.F. (1998). Floral dip: A simplified method for Agrobacterium-mediated transformation of *Arabidopsis thaliana*. Plant J..

[42] Welti R., Li W., Li M., Sang Y., Biesiada H., Zhou H.E., Rajashekar C.B., Williams T.D., Wang X. (2002). Profiling membrane lipids in plant stress responses. Role of phospholipase D alpha in freezing-induced lipid changes in *Arabidopsis*. J. Biol. Chem..

[43] Li B., Li L., Li M., Lam S.M., Wang G., Wu Y., Zhang H., Niu C., Zhang X., Liu X. (2019). Microbiota Depletion Impairs Thermogenesis of Brown Adipose Tissue and Browning of White Adipose Tissue. Cell Rep..

