# Supplementary material for: R2R3 MYB Transcription Factor GhMYB201 Promotes Cotton Fiber Elongation via Cell Wall Loosening and Very-Long-Chain Fatty Acid Synthesis
Source: Int J Mol Sci. 2024 Sep 3;25(17):9559. doi: 10.3390/ijms25179559 (PMC11395274; doi:10.3390/ijms25179559)
Supplement: Supplementary file 1 [file ijms-25-09559-s001.zip › ijms-3186323-supplementary.pdf]

Supplemental Table S1. Transcription factors highly enriched in elongating fibers

Gene_id	Annotation	Average-ef (FPKM)[a]	Average-all (FPKM)[b]	Enrichment Fold[c]	Enrichment Factor[d]
**bZIP**					
**Gh_A01G0419**	bZIP	22.57	7.13	3.16	71.41
**Gh_D01G0423**	bZIP	38.94	10.91	3.57	138.96
**Gh_A13G1269**	bZIP	49.75	9.09	5.47	272.26
**Gh_D13G1573**	bZIP	39.59	10.3	3.84	152.23
**homeobox**					
**Gh_A09G0391**	HDG	28.7	4.98	5.76	165.29
**Gh_Sca007830G01**	HDG	46.36	11.24	4.12	191.16
**Gh_D10G0270**	HOX3	15.76	2.83	5.57	87.83
**Gh_D12G2590**	HDG11	22.66	6.18	3.67	83.07
**bHLH**					
**Gh_A01G2041**		29.51	6.27	4.71	138.96
**Gh_A09G1863**	bHLH	7.22	0.88	8.23	59.45
**Gh_D04G0454**	bHLH	12.02	2.09	5.76	69.21
**GATA ZF**					
**Gh_A03G1598**	GATA ZF	56.94	10.91	5.22	297.33
**Gh_A12G2074**	GATA ZF	109.97	25.09	4.38	482.06
**Gh_D12G2250**	GATA ZF	42.75	10.91	3.92	167.52
**Gh_D12G2631**	GATA ZF	64.3	14.36	4.48	288.02
**Gh_A13G0161**	GATA ZF	92.32	20.79	4.44	409.95
**Gh_A13G0162**	GATA ZF	160.93	44.22	3.64	585.64
**MYB**					
**Gh_A04G1024**	MYB 73	23.38	5.74	4.07	95.24
**Gh_A05G3123**	MYB 66	18.72	4.93	3.8	71.08
**Gh_A09G2380**	MYB 85	40.94	10.03	4.08	167.12
**Gh_A13G1399**	MYB 201	108.03	15.95	6.77	731.74
**Gh_D13G1712**	MYB 201	188.39	27.31	6.9	1299.4
**Gh_A13G2154**	MYB 106	103.62	29.75	3.48	360.86
**Gh_A10G0338**	MYB-like	39.04	7.28	5.36	209.41
**Gh_D10G0344**	MYB-like	36.73	7.31	5.03	184.63
**Others**					
**Gh_A04G0810**	GRAS	9.95	1.86	5.35	53.26
**Gh_A05G1526**	NAC	24.51	7.92	3.09	75.82
**Gh_A11G0279**	TCP 14	25.1	7.05	3.56	89.32

Note: a, the average fragments per kilobase per million mapped reads (FPKM) value in fibers of 5 and 10 DPA;
b, the average FPKM value in 20 tissues including fibers of 5, 10, 20 and 25 DPA, ovules of -3, -1, 0, 1, 3, 5, 10, 20, 25 and 35 DPA, roots, stems, leaves, petals, stamens and pistils.
c, Enrichment fold=Average-ef (FPKM)/Average-all (FPKM).
d, Enrichment factor=Enrichment fold × Average-ef (FPKM).

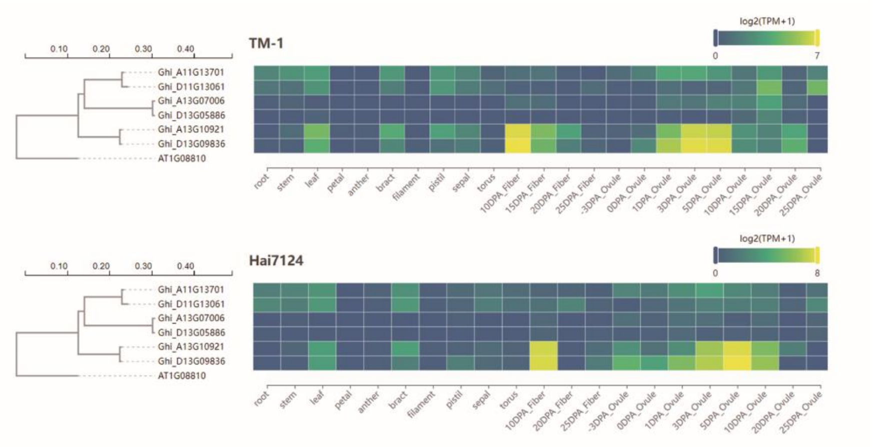

Supplemental Figure S1. Transcript levels of AtMYB60 homologous genes in various cotton tissues
Heatmap indicates fragments per kilobase per million mapped reads (FPKM) values of six AtMYB60 homologous genes (*Ghi_A11G13701/GhMYB105-At*, *Ghi_D11G13061/GhMYB105-Dt*, *Ghi_A13G07006/GhMYB192-At*, *Ghi_D13G05886/GhMYB192-Dt*, *Ghi_A13G10921/GhMYB201-At*, and *Ghi_D13G09836/GhMYB201-Dt*) in various tissues from *G. hirsutum* acc. TM-1 [1]. Tissues include fibers (Fi) and ovules (Ou) of various DPA, petal (Peta), pistil (Pist), root, stamen (Stamen), stem and leaf.

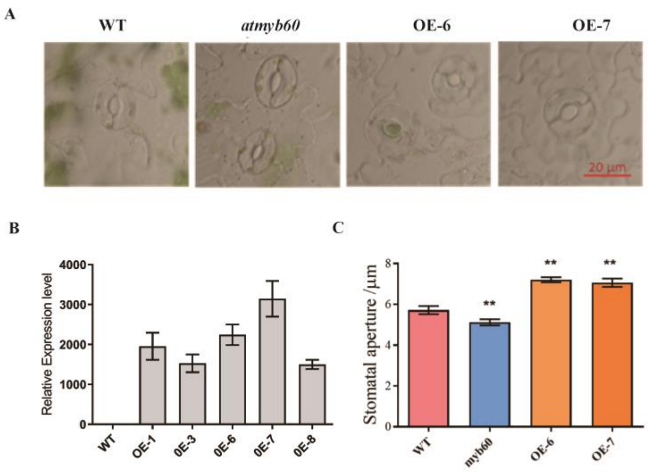

Supplemental Figure S2. *GhMYB201* rescues the regulation of stomatal aperture in Arabidopsis *atmyb60* mutant

A, The stomatal aperture of wild type, *atmyb60* mutant (SALK_013126C) and *GhMYB201* over-expressing Arabidopsis lines (OE). Bar = 20 μm. B, RT-qPCR analysis of *GhMYB201* transcript levels in transgenic Arabidopsis lines. Mean values and standard errors were calculated from three biological replicates. C, Statistical analysis of stomatal aperture in A. Data are means ±SEM, n > 100 stomata.

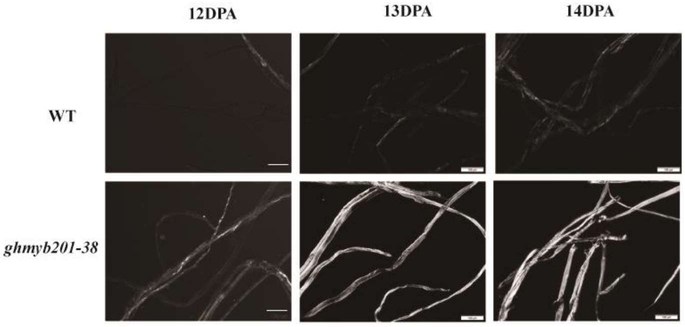

Supplemental Figure S3. Birefringence microscopy of Cellulose deposition in developing fibers
WT and *ghmyb201-38* fibers were observed at 12, 13 and 14 DPA fibers. Scale bar = 100 μm.

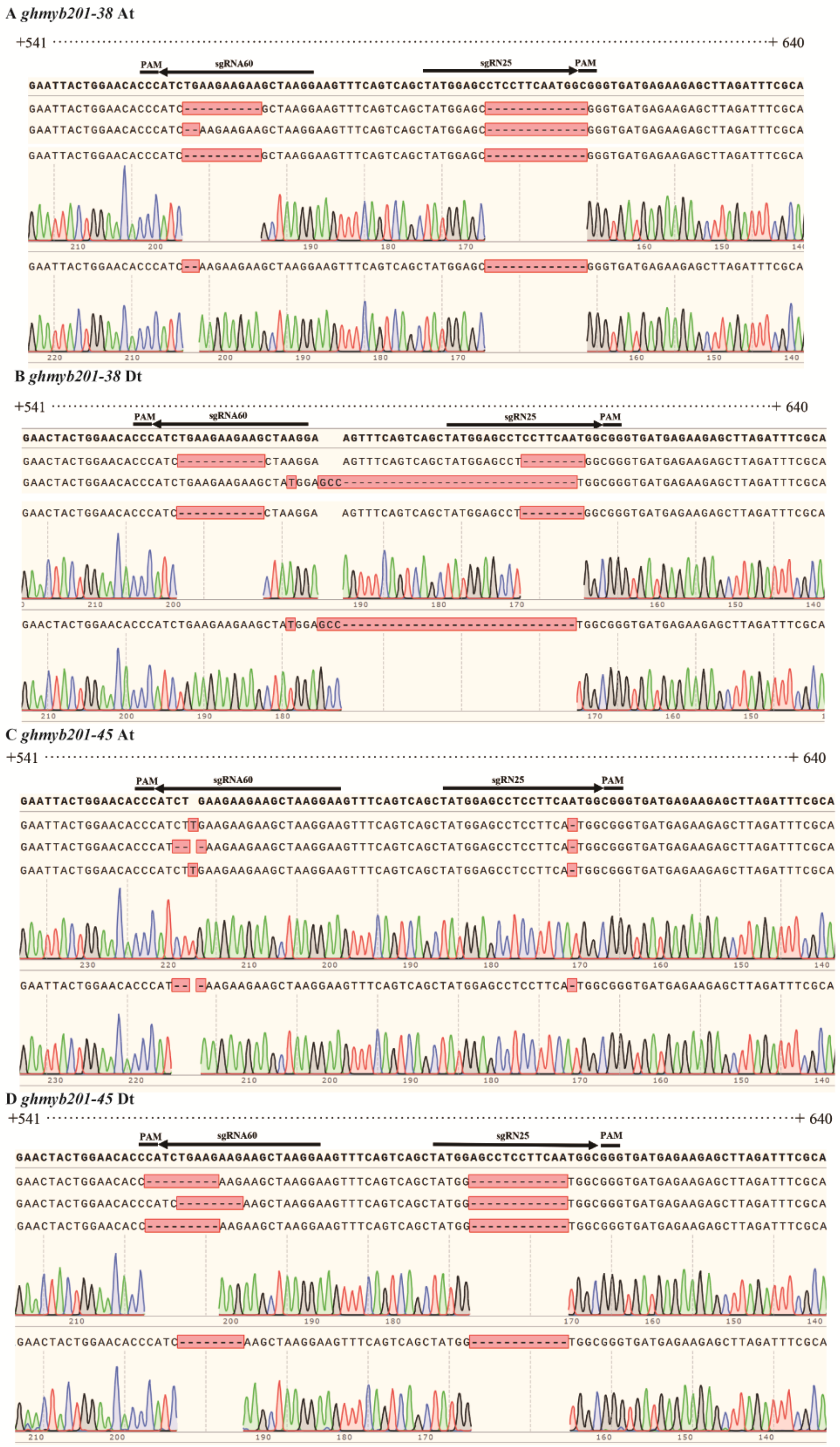

Supplemental Figure S4 Characterization of GhMYB201 plants generated by CRISPR/Cas9 gene editing

A-B *ghmyb201-38* genomic sequences of target sites from T1 plants, obtained by Sanger sequencing. The mutation type of *ghmyb201-38* knockout line with 2 sgRNAs. C-D *ghmyb201-45* genomic sequences of target sites from T1 plants, obtained by Sanger sequencing. The mutation type of *ghmyb201-45* knockout line with 2 sgRNAs.

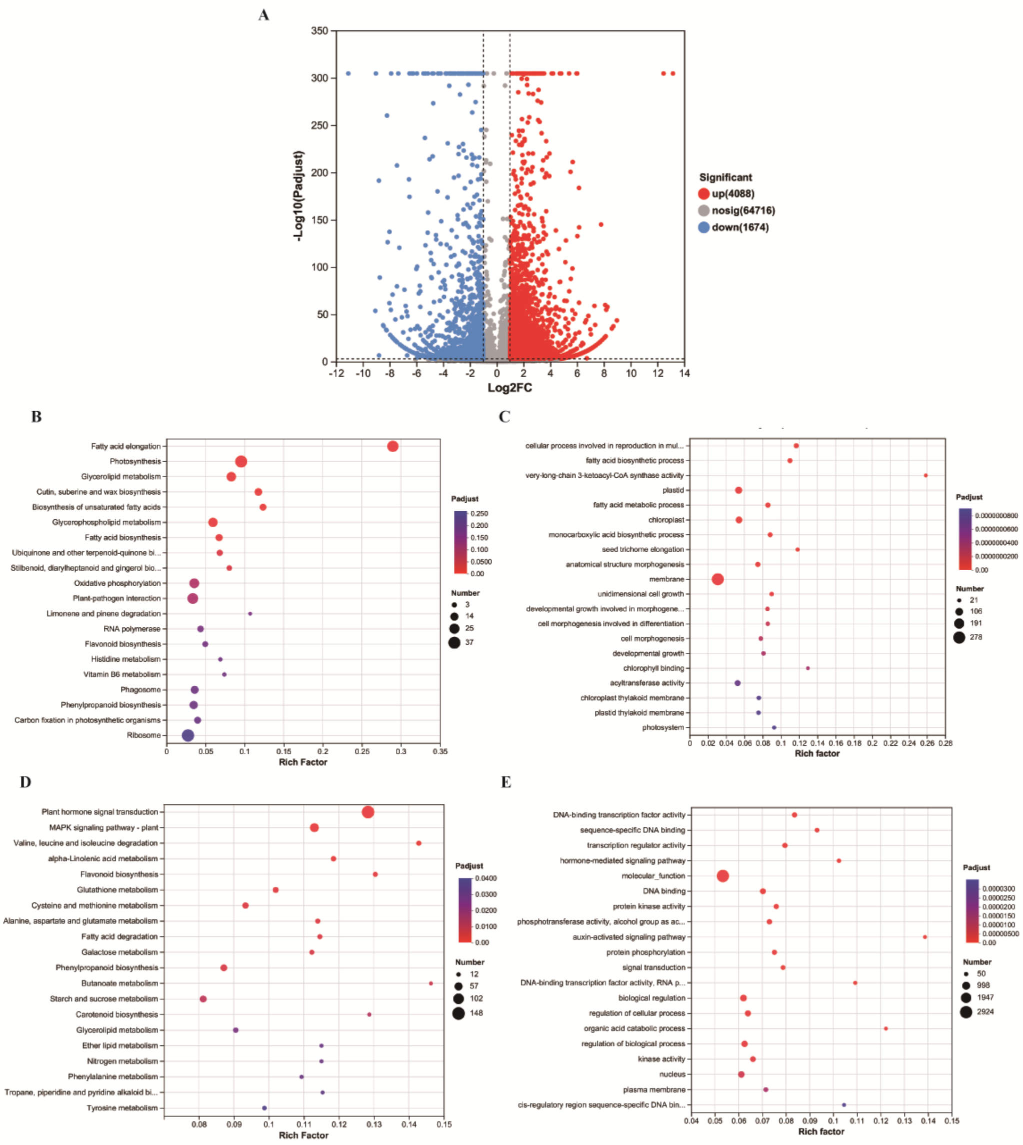

Supplemental Figure S5. RNA-Seq analysis of 7-DPA fibers of *GhMYB201* knockout line (*ghmyb201-38*) and wild type

(A) Volcano map of the differentially expressed genes (DEGs). Blue and red dots represent the down- and up-regulated genes in *GhMYB201* knockout fibers, compared with the wild type, respectively. (B) Enriched KEGG pathways in downregulated DEGs. (C) Enriched GO pathways in downregulated DEGs. (D) Enriched KEGG pathways in upregulated DEGs. (E) Enriched GO pathways in upregulated DEGs.

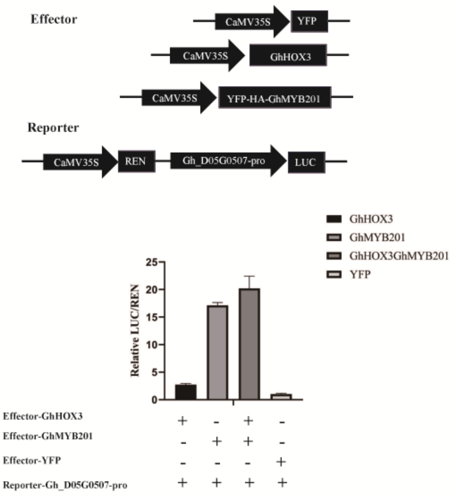

Supplemental Figure S6. Transcriptional regulation of target gene by GhMYB201 and the effect of GhHOX3

1. Zhang, T.; Hu, Y.; Jiang, W.; Fang, L.; Guan, X.; Chen, J.; Zhang, J.; Saski, C.A.; Scheffler, B.E.; Stelly, D.M.; et al. Sequencing of allotetraploid cotton (Gossypium hirsutum L. acc. TM-1) provides a resource for fiber improvement. *Nat Biotechnol* **2015**, *33*, 531-537, doi:10.1038/nbt.3207.
